# JDart: Dynamic Symbolic Execution for Java Bytecode (Competition Contribution)

**DOI:** 10.1007/978-3-030-45237-7_28

**Published:** 2020-03-13

**Authors:** Malte Mues, Falk Howar

**Affiliations:** 8 Johannes Kepler University, Linz, Austria; 9 University of Birmingham, Birmingham, UK; Dortmund University of Technology, Dortmund, Germany

## Abstract

JDart performs dynamic symbolic execution of Java programs: it executes programs with concrete inputs while recording symbolic constraints on executed program paths. A constraint solver is then used for generating new concrete values from recorded constraints that drive execution along previously unexplored paths. JDart is built on top of the Java PathFinder software model checker and uses the JConstraints library for the integration of constraint solvers.

## 1 Overview

JDART is a dynamic symbolic execution engine for the JVM build on top of Java PathFinder (JPF) [11]. Dynamic symbolic execution [4,6] (sometimes also referred to as concolic execution) executes programs with concrete values while recording symbolic constraints for execution paths. The approach combines the benefits of fast concrete execution with the possibility of generating new concrete values, triggered by symbolic constraints, that exercise previously unexplored program behaviors. JDART can be used for checking assertions in Java programs: Concolic execution will explore new program paths until either (a) an assertion violation is discovered, (b) all program paths have been explored, or (c) resource limits of the analysis are exhausted.

The initial driver of the development of JDART was the need for an analysis that is robust enough to handle large and complex systems, concretely the AUTORESOLVER software for prediction and resolution of airplane loss of separation developed at NASA Ames Research Center [7]. Though JDART provides a robust and scalable platform for dynamic symbolic analysis of JAVA programs [7], we had to extend its functionality in several ways in order to be able to compete at SV-COMP 2020 [1]. We developed:

1. a new analysis mode in which fresh symbolic variables are introduced during analysis (in contrast to a fixed number of manually declared symbolic values),
2. a number of symbolic models encoding environment behavior (driven by SV-COMP 2020 benchmarks), and
3. a new mode for solving constraints in a sequence of attempts using successively weaker bounds on variables (cf. Section 2).

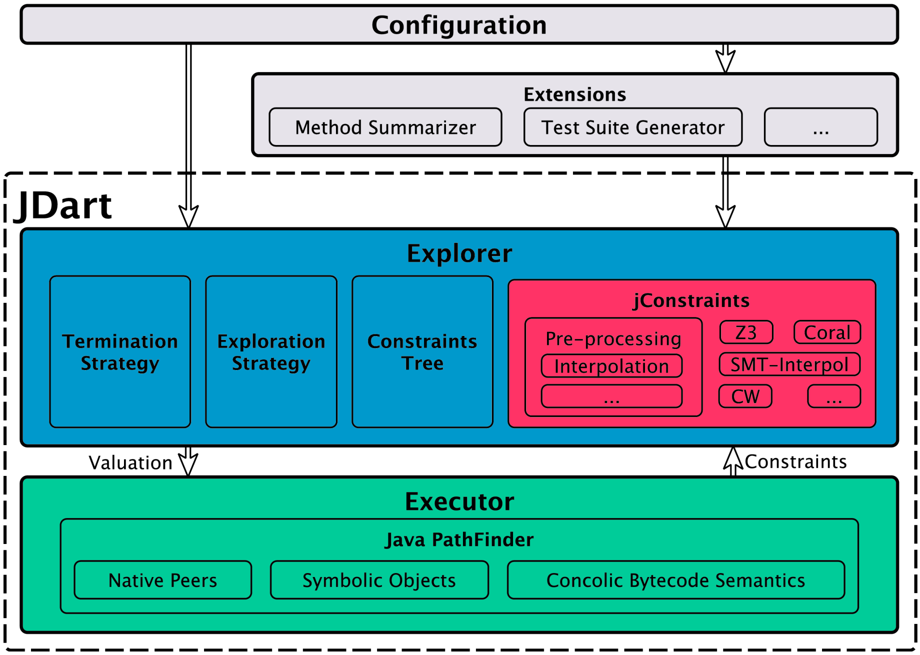

Fig. 1: Architecture of JDart [7].

While (1) enabled JDart to enter the competition, (2) accounts for the largest part of improvements over our own baseline, and (3) contributes to better performance on some benchmarks with assertion violations in big state spaces.

## 2 Architecture

JDart combines dynamic execution with recording and analysis of symbolic path constraints. It runs as an extension of the JPF software model checker [11]. In particular, JDart uses the Java virtual machine implemented by JPF and its capabilities for annotating values on the stack and the heap with symbolic information. The tool itself is written in Java and uses JConstraints [5] for encoding SMT problems. Moreover, JConstraints acts as a frontend to an SMT solver (e.g., Z3 [3]) used for finding concrete values that drive the analysis.

Figure 1 illustrates the architecture of JDart: The tool consists of three layers: Concrete analysis frontends make up the top layer (e.g., generation of method summaries, generation of test suites, assertion checking). The main components record and analyze execution paths (Explorer) and perform concolic execution (Executor). The *Executor* uses concolic implementations of bytecode instructions. These bytecodes are executed instead of the original JPF bytecodes. A concolic bytecode tracks the symbolic representation of a value and annotates a concrete value with its symbolic counterpart. Whenever execution takes a branching decision based on a concrete value with a symbolic annotation, the symbolic value is added to the constraints tree maintained by the *Explorer*. A constraint solver is used for finding concrete values that drive execution along unexplored paths of the tree.

Leveraging the modular architecture of JDart and JConstraints, we implemented a meta-constraint solver for finding small concrete values for symbolic numeric variables. This allows JDart to find assertion violations faster and with less resource consumption in cases where a symbolic variable controls the number or length of execution paths (e.g., symbolic array size or a symbolic loop bound). The meta-constraint solver performs multiple calls to an SMT solver, adding successively weaker bounds to numeric variables. E.g., for a path constraint $\varphi$ over symbolic numeric variable $x$, the solver adds bounds $(-z \leq x) \wedge (x \leq z)$ with $z \in (1,\ 2,\ 3,\ 5,\ 8,\ 13,\ 21,\ \ldots)$, i.e., the first numbers in the Fibonacci sequence. If the solver finds a model for the constraint, JDart uses this model for driving concolic execution. In case no model is found in a fixed number of attempts, the SMT solver is called without added bounds. The number of attempts is a configuration parameter of JDart and was fixed to 7 for SV-COMP 2020.

Analysis of JDart can be bounded by termination strategies. When checking assertions the termination strategy is stopping on the first occurrence of an assertion violation. Additional strategies could be bounding depth of the symbolic analysis, bounding runtime, or termination on specific errors. We refer the reader to [7] for a more detailed and complete discussion of the features of JDart.

## 3 Strengths and Weaknesses

JDart scored 524 points (max. of 602) in the Java track and was declared third winner for Java, behind JBMC (527 points) [2] and Java Ranger (549 points) [9]. All other tools scored considerably fewer points than JDart (next best is COASTAL [10] with 472). As Java Ranger and JBMC, JDart did not report a single incorrect verdict. JDart exhibits the general strengths and weaknesses of dynamic and symbolic analysis approaches for Java programs:

**Runtime.** Driven by concrete execution, the analysis is fairly fast. JDart is overall the second fastest tool in cases where it can provide an answer. Not using bounds JDart, on the other hand, has a relatively high number of timeouts and runs that terminate due to resource limitations — and thus only the fourth lowest cumulative runtime.

**Symbolic Strings.** Particular to Java verification is the challenge of providing models for the behavior of classes in the Java standard library. In SV-COMP 2020 such models are mostly required for analyzing benchmarks that extensively incorporate String processing. We made a substantial contribution to the code base of JDart and implemented models for `java.lang.String` and related classes. As a consequence, JDart can analyze all but one corresponding benchmark examples (JDart currently cannot analyze regular expressions symbolically).

**Unbounded Behavior.** Based on principles of symbolic execution, JDart does not terminate on unbounded loops or in case of unbounded recursion, leading to a number of timeouts on the corresponding set of benchmarks.

## 4 Tool Setup

The source code of JDart used for the competition artifact [8] is available on GitHub[1]. JDart is designed as a plug-in to JPF and relies on ant as a build system. One of its dependencies is the `jpf-core` project [11]. The other dependency is the JConstraints library, which was configured to use Z3 [3] with incremental solving as a constraint solver for SV-COMP 2020.

For the competition, JDart is wrapped by the `run-jdart.sh` shell script which generates `.jpf` configuration files, specifying which benchmark to analyze and the global configuration options to JDart: For SV-COMP 2020 all termination criteria except for assertion violations are disabled, executing JDart as an almost unbounded assertion checker (the only bound in place is an upper bound of 127 on maximal length of String variables). The shell script records and interprets the output of JDart and can also report the version of JDart.

## 5 Software Project

The version of JDart that was used in SV-COMP 2020 is maintained by the Automated Quality Assurance Group at Technical University of Dortmund (in particular by the authors of this paper) and is available under the Apache License, version 2.0, on GitHub[1]. An initial version of JDart was developed by the authors of [7] at NASA Ames Research Center and Carnegie Mellon University. The original version of JDart is available on GitHub[2].

**Acknowledgments.** We are grateful for the work on JDart and JConstraints by the respective original authors. Our success would not have been possible without their contributions.

## References

[1] Beyer, D.: Advances in automatic software verification: SV-COMP 2020. In: Proc. TACAS (2). LNCS 12079, Springer (2020), https://www.sosy-lab.org/research/pub/2020-TACAS.Advances_in_Automatic_Software_Verification_SV-COMP_2020.pdf

[2] Cordeiro, L., Kroening, D., Schrammel, P.: Jbmc: Bounded model checking for java bytecode. In: Beyer, D., Huisman, M., Kordon, F., Steffen, B. (eds.) Tools and Algorithms for the Construction and Analysis of Systems. pp. 219–223. Springer International Publishing, Cham (2019). 10.1007/978-3-030-17502-3_17

[3] De Moura, L., Bjørner, N.: Z3: An efficient smt solver. In: International conference on Tools and Algorithms for the Construction and Analysis of Systems. pp. 337–340. Springer (2008). 10.1007/978-3-540-78800-3_24

[4] Godefroid, P., Klarlund, N., Sen, K.: Dart: Directed automated random testing. In: Proceedings of the 2005 ACM SIGPLAN Conference on Programming Language Design and Implementation. pp. 213–223. PLDI ’05, ACM (2005). 10.1007/978-3-642-19237-1_4

[5] Howar, F., Jabbour, F., Mues, M.: JConstraints: A library for working with logic expressions in Java. In: Models, Mindsets, Meta: The What, the How, and the Why Not?, pp. 310–325. Springer (2019). 10.1007/978-3-030-22348-9_19

[6] King JC (1976). Symbolic execution and program testing. Commun. ACM.

[7] Luckow, K.S., Dimjasevic, M., Giannakopoulou, D., Howar, F., Isberner, M., Kahsai, T., Rakamaric, Z., Raman, V.: JDart: A dynamic symbolic analysis framework. In: Proceedings of TACAS 2016. pp. 442–459 (2016). 10.1007/978-3-662-49674-9_26

[8] Mues M, Howar F (2020). JDart artifact used in SV-COMP 2020. Zenodo.

[9] Sharma, V., Hussein, S., Whalen, M., McCamant, S., Visser, W.: Java Ranger at SV-COMP 2020 (competition contribution). In: Biere, A., Parker, D. (eds.) TACAS 2020. LNCS, vol. 12079, pp. 393–397. Springer, Cham (2020). 10.1007/978-3-030-45237-7_27

[10] Visser, W., Geldenhuys, J.: COASTAL: Combining concolic and fuzzing for Java (competition contribution). In: Biere, A., Parker, D. (eds.) TACAS 2020. LNCS, vol. 12079, pp. 373–377. Springer, Cham (2020). 10.1007/978-3-030-45237-7_23

[11] Visser, W., Havelund, K., Brat, G., Park, S., Lerda, F.: Model checking programs. Automated Software Engineering **10**(2), 203–232 (Apr 2003). 10.1023/A:1022920129859

